# High circulating HMGB1 indicates good prognosis in patients with advanced leiomyosarcoma under chemoimmunotherapy

**DOI:** 10.1080/2162402X.2024.2432059

**Published:** 2024-11-21

**Authors:** Lucillia Bezu, Guido Kroemer

**Affiliations:** a Equipe labellisée par La Ligue contre le cancer, Université de Paris Cité, Sorbonne Université, Paris, France; b Metabolomics and Cell Biology Platforms, Gustave Roussy, Villejuif, France; c Département d’Anesthésie, Chirurgie et Interventionnel, Gustave Roussy, Villejuif, France; d EuroPeriscope, ESA-IC Onco-Anesthesiology Research Group (ESAIC_RG_EP), Brussels, Belgium; e Pôle de Biologie, Hôpital européen Georges Pompidou, AP-HP, Paris, France

**Keywords:** Biomarker, cancer, chemotherapy, immunotherapy, PD-1 blockade

## Abstract

Few clinical studies investigated the putative link between the activation of immunogenic cell death (ICD) and the oncological outcome. Recent data, published in a Phase 1b trial, demonstrated that an ICD-associated surge in the plasma concentration of high-mobility group box 1 (HMGB1) indicates favorable prognosis in patients with advanced leiomyosarcomas treated with the combination of doxorubicin, dacarbazine and nivolumab.

## Main Text

Preclinical data indicate that some anticancer drugs are capable of triggering a particular modality of cell death called immunogenic cell death (ICD) that is characterized by the induction of T-cell-mediated anticancer immune responses. It is important to note that only few particularly successful cytotoxicants are endowed with the capacity of ICD induction; most are not. After treatment with ICD-inducing chemotherapeutics such as doxorubicin, dying tumor cells emit specific danger-associated molecular patterns (DAMPs). These DAMPs include extracellular ATP, which causes the recruitment of myeloid cells including dendritic cells (DCs) into the vicinity of cancer undergoing ICD, calreticulin (CALR), which translocates to the plasma membrane of cancer cells to stimulate their uptake by DCs, as well as the release of high-mobility group box 1 (HMGB1), which stimulates the maturation and DCs by an action of toll-like receptor 4 (TLR4). Of note, CALR exposure is obligatorily preceded by the phosphorylation of eukaryotic initiation factor 2α (eIF2α), which can be measured by immunohistochemistry. As a final outcome, DCs present tumor antigens to cytotoxic T lymphocytes, which are primed to synthetize interferon-γ and to kill residual malignant cells, and later generate memory T cells to offer durable disease control.^1^

Therapeutic induction of ICD has the property to convert initially ‘cold’ cancers that lack a local immune infiltrate indicating immunosurveillance into ‘hot’ cancers that exhibit the stigmata of T cell-mediated cancer cell elimination. Accordingly, ICD-inducing chemotherapeutics have been incorporated into established treatment protocols to improve their efficacy, notably in the context of immunotherapy with immune checkpoint inhibitors targeting CTLA-4, PD-1, and PD-L1.^2^ In addition, it has been reported in some clinical trials that ICD-associated DAMPs assessed in the blood or in tumor biopsies from patients could serve as predictive and prognostic markers. While CALR exposure on cancer cells positively correlates with overall and progression-free survival (PFS), elevated HMGB1 levels has been associated with poor outcomes in some trials (Table 1). In fact, the role of HMGB1 in cancer is complex. Many mechanisms increase the HMGB1 plasma level during oncogenesis; for instance, in the context of local or systemic inflammation. In the context of ICD-inducing therapies, elevated circulating HMGB1 concentrations associated with other DAMPs correlate with positive outcome.^3,5,6,8^ In contrast, elevations of HMGB1 without that of other DAMPs apparently are not sufficient to trigger efficient cancer immunosurveillance after treatment with chemotherapeutics such as taxanes, carboplatin, or gemcitabine that must be classified as partial rather than full ICD inducers.^4,9^ Thus, a surge in plasma HMGB1 induced by partial ICD inducers may reflect cancer progression rather than a successful therapeutic manipulation^7^ (Table 1). In conclusion, increases in circulating HMGB1 levels must be interpreted with caution in a context-dependent fashion.

**Table 1.** Studies investigating the correlation between HMGB1 and oncological outcomes after treatment with partial (*p*) or full (*f*) immunogenic cell death inducers.

Cancers	Patients (n)	Treatment	Outcomes	Ref
Breast	41	Epirubicin **(*f*)** + docetaxel **(*p*)**	Increased HMGB1 in plasma is correlated with favorable therapeutic responses	3
Breast	58	Paclitaxel **(*p*)** or docetaxel **(*p*)** + bevacizumab	No correlation between HMGB1 level and PFS/OS	4
Esophagus	88	5-FU/CDDP/docetaxel **(*p*)** + radiotherapy **(*f*)**	Increased HMGB1 in plasma is correlated with improved OS	5
HNSCC	11	5-FU/CDDP/mitomycin **(*p*)** + radiotherapy **(*f*)**	Increased HMGB1 is correlated with enhanced PFS	6
Pancreas	78	-Gemcitabine **(*p*)** ± erlotinib or everolimus -Capecitabine ± erlotinib or axitinib -Nab-paclitaxel **(*p*)**	Increased HMGB1 in plasma is correlated with worse PFS/OS	7
Rectum	50	-Oxaliplatin **(*f*)** +5-FU + radiotherapy **(*f*)** -Oxaliplatin **(*f*)** + capecitabine + radiotherapy **(*f*)**	HMGB1 is correlated with better PFS and OS	8
Solid tumors	52	-Carboplatin **(*p*)** + pemetrexed + pembrolizumab -Pembrolizumab -Cetuximab	No variation of HMGB1 level after treatment. No difference in OS/PFS between patients with increased or decreased HMGB1	9

Abbreviations: CDDP, cisplatin; 5-FU, 5-fluorouracil; HMGB1, high mobility group box 1; HNSCC, head and neck squamous cell carcinoma; PFS, progression-free survival; OS, overall survival.

Recently, Martin-Broto et al. demonstrated in a Phase 1b clinical trial that combined treatment with a triad of molecules, namely, (i) the established ICD inducer doxorubicin, (ii) the alkylating agent dacarbazine and (iii) the anti-PD1 antibody nivolumab, is well tolerated by patients with advanced leiomyosarcoma. Among the 23 treated patients, 18 blood samples were available, 78% of which showed an increased in HMGB1 at 6 weeks after treatment. Moreover, the 10 patients exhibiting an increase in circulating HMGB1 above the threshold of 12% had a significantly (*p* = 0.008) longer PFS (12.6 months with a 95% confidence interval [CI] of 10.6 to 14.6) than the 8 patients exhibiting a lower HMGB1 levels (7 months with a 95% CI of 5 to 8.9). Based on these results, the authors concluded that the triple combination would exert a synergistic therapeutic effect and that HMGB1 serves as a favorable prognostic marker. However, it is worth noting that 50% of patients without progression at the moment of data analysis (and with the HMGB1 level available) had HMGB1 increases of <12%, and 50% of patients with progression during chemo-immunotherapy displayed HMGB1 modifications of >12%, shedding doubts on the correlation between HMGB1 levels and PFS.^10^

**Figure 1.**
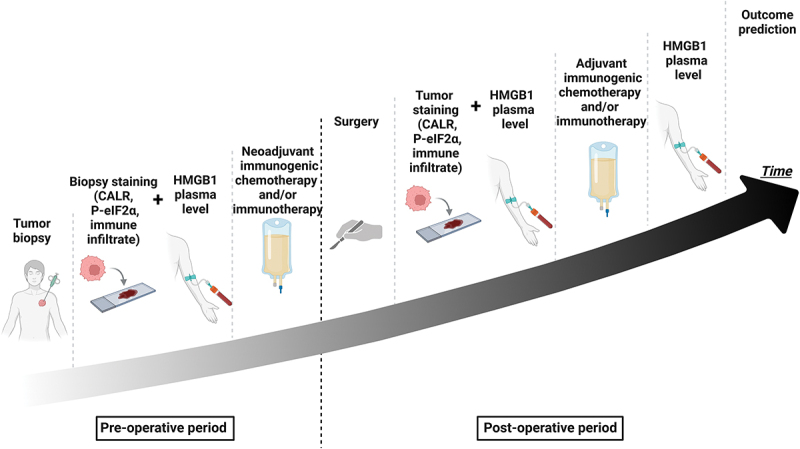
Proposed protocol for assessing the hallmarks of immunogenic cell death in oncological practice. Increased HMGB1 plasma level combined with increased calreticulin exposure and eIf2α phosphorylation in tumor samples after chemotherapy, chemoimmunotherapy, or immunotherapy might constitute predictive markers of better prognosis, especially if the treatment elicits signs of an anticancer immune responses reflected by the T cell infiltrate of the tumors. *CALR, calreticulin, HMGB1, high mobility group box 1, P-eIf2α, phosphorylated eukaryotic translation initiation factor 2A. Created with www.BioRender.com.*

HMGB1 is not only linked to ICD but can also be increased in several stress conditions such as therapy-related inflammation and neoplastic disease as such. Combining the assessment of HMGB1 concentrations in patient plasmas with other ICD-relevant parameters such as CALR expression and eIF2α phosphorylation in tumor biopsies should inform more accurately on the anticancer immune response (Figure 1). Of note, two leiomyosarcoma patients enrolled in the aforementioned trial^10^ received additional treatments with docetaxel or radiotherapy, both of which might promote ICD and cause an elevation of circulating HMGB1 concentrations. Furthermore, it would have been interesting to compare the baseline characteristics of patients with or without increased HMGB1 of >12% to understand longitudinal variations in HMGB1 levels on a patient-by-patient basis. Finally, a multivariate analysis should have been carried out to jointly evaluate the prognostic impact of HMGB1 concentrations, PD-L1 expression, and other outcome-related factors such as tumor size and TNM stage. Unfortunately, the small number of patients precluded any in-depth analysis of this kind.

In conclusion, despite encouraging trends, it would be premature to interpret increased HMGB1 as an unequivocal sign of good prognosis in patients with advanced leiomyosarcoma treated with doxorubicin, dacarbazine and nivolumab. Further biological and statistical analyses of larger validation trials are necessary to confirm this conclusion. It will be particularly interesting to see whether other forms of sarcoma will respond to the combination treatment in a similar fashion.
